# Supplementary material for: Stratification of COVID-19 Severity Using SeptiCyte RAPID, a Novel Host Immune Response Test
Source: Viruses. 2023 Feb 2;15(2):419. doi: 10.3390/v15020419 (PMC9960895; doi:10.3390/v15020419)
Supplement: Supplementary file 1 [file viruses-15-00419-s001.zip › viruses-2178112-supplementary.pdf]

Stratification of COVID-19 severity using SeptiCyte RAPID, a novel host immune response test

Victor Gravrand[1], François Mellot[2], Felix Ackerman[3], Maric Christine Ballester[4], Benjamin Zuber[5], James T. Kirk[6], Krupa Navalkar[6], Thomas D. Yager[6], Fabien Petit[1], Tiffany Pascreau[1,7], Eric Farfour[1], Marc Vasse [1,7, *]

[1] Biology Department, Foch Hospital, Suresnes 92150, France

[2] Radiology Department, Foch Hospital, Suresnes 92150, France

[3] Internal Medicine Department, Foch Hospital, Suresnes 92150, France

[4] Emergency Department, Foch Hospital, Suresnes 92150, France

[5] Intensive Care Unit, Foch Hospital, Suresnes 92150, France

[6] Immunexpress, Inc., Seattle, WA 98109, USA

[7] Institut National de la Santé et de la Recherche Médicale (INSERM), UMR-S1176 , Le Kremlin-Bicêtre 94270, France

* Correspondence: m.vasse@hopital-foch.com; Tel.: +33-(0)1-46-25-29-57

Supplementary Tables

Supplementary Table S1: Patient comorbidities and treatments

Characteristic	Stratum	N (%)
Number of comorbidities	5	1 (1.0%)
	4	5 (5.3%)
	3	13 (13.8%)
	2	30 (31.9 %)
	1	23 (24.5.0%)
	0	22 (23.4 %)
Types of comorbidities	None listed	22 (23.4%)
	Hypertension	42 (44.7%)
	Diabetes	32 (34.0 %)
	Obesity (BMI ≥ 30)*	22 (23.4%)
	Pulmonary	15 (16.0%)
	Renal	10 (10.6%)
	Cardiovascular	13 (13.8 %)
	Cancer	10 (10.6%)
	Dyslipidemia	6 (6.4 %)
	Hypothyroidism	5 (5.3%)
Treatments	ICU	30 (31.9%)
	Corticosteroids	51 (54.3%)
	Tocilizumab	24 (25.5 %)
	Oxygen	57 (60.6%)
	Intubation	7 (7.4%)
	ECMO	3 (3.2%)
	Antibiotics	36 (38.3%)

*BMI available for only 46 patients

Supplementary Table S2 : Distribution of SARS-CoV-2 (+) patients across COVID-19 severity categories and hospital locations. COVID-19 severity assessments were made on the basis of chest CT scans, conducted within 24 hours of SeptiCyte RAPID measurement. Range is specified in [ ] square brackets, and percentage is specified in ( ) parentheses. The median values for age and the median time between onset of symptoms and SeptiCyte result are reported for those patients with CT scan results.

	Emergency Department (Fig 1a) (n=67 total; 49 with CT scan results)					ICU (Fig 1b)	Conventional Unit,Viremia (Fig. 1c)
Follow-up	Discharged (< 24 hours in ED)	Conventional Hospitalization	Delayed ICU admission	Immediate ICU admission	Deceased in ED		
n (total) = 94	25	33	4	3	2	23	4
n (with CT scan result)	12	30	4	2	1	15	4
F/M (with CT scan result)	6/6	13/17	2/2	0/2	1/0	6/9	1/3
Median age, years	58 [20 – 84]	71 [23 – 96]	66 [63 – 66]	77 [76 – 78]	85	59 [27 – 78]	57 [38 – 67]
Median time between onset of symptoms and SeptiCyte result, days (symptomatic patients)	7 (n=11) [1 – 12]	7 (n=28) [0 – 30]	6 (n=4) [4 – 9]	8.5 (n=2) [7 – 10]	NA	10 [1 – 24]	9 [9 – 11]
Radiologic injury, n (%)							
Absence (0%)	5 (20%)	1 (3%)	0	0	0	0	0

	Emergency Department (Fig 1a) (n=67 total; 49 with CT scan results)					ICU (Fig 1b)	Conventional Unit,Viremia (Fig. 1c)
Follow-up	Discharged (< 24 hours in ED)	Conventional Hospitalization	Delayed ICU admission	Immediate ICU admission	Deceased in ED		
Mild (present but <10 %)	1 (4%)	8 (24%)	0	0	0	0	1 (25%)
Moderate [10 – 25 %]	4 (16%)	8 (24%)	1 (25%)	0	1 (50%)	2 (8.7%)	1 (25%)
Extensive (25 - 50 %)	1 (4%)	11 (33%)	1 (25%)	1 (33.3%)	0	4 (17%)	2 (50%)
Severe [50 – 75 %]	0	1 (3%)	1 (25%)	1 (33.3%)	0	6 (26%)	0
Critical (> 75 %)	0	0	0	0	0	3 (13.1%)	0
Not specific	1 (4%)	1 (3%)	1 (25%)	0	0	0	0
Not performed	13 (52%)	3 (9.1%)	0	1 (33.3%)	1 (50%)	8 (34.8%)	0

Supplementary Table S3: Logistic Regression models (see Figures. 4A, 4B in main text). A complete permutation analysis of different logistic combinations of up to six variables (SeptiScore, monocytes, CD16-monocytes, D-dimer, lactate and CRP) was conducted, resulting in 63 independent logistic regression models. The models were used to discriminate Critical + Severe in ICU (n=13) vs. Moderate + Mild not in ICU (n = 24) and their AUC values were calculated. Interquartile ranges for AUC were obtained by resampling from 100 replicate datasets. Missing values in the line data were imputed using Amelia, a multiple imputation algorithm [Reference 15 in main text]. Multiple imputation is claimed to have reduced bias and increased accuracy over point-imputation methods such as "mean" or "median" imputation [Reference 15 in main text]. Abbreviations: CRP, C-reactive protein; Mono, monocytes; Mono.CD16, CD16-monocytes.

Model	variables	MEAN	MEDIAN	25%	75%
1	CRP	0.867	0.871	0.860	0.890
2	D-dimer	0.630	0.640	0.576	0.679
3	Lactate	0.668	0.680	0.607	0.729
4	Mono	0.725	0.722	0.703	0.748
5	Mono.CD16	0.750	0.752	0.698	0.791
6	D-dimer + Lactate	0.663	0.674	0.613	0.724
7	Lactate + Mono	0.739	0.742	0.716	0.761
8	D-dimer + Lactate + Mono	0.767	0.765	0.739	0.792
9	Mono + Mono.CD16	0.768	0.758	0.734	0.807
10	D-dimer + Mono	0.769	0.769	0.742	0.792
11	Lactate + Mono.CD16	0.779	0.778	0.730	0.838
12	Lactate + Mono + Mono.CD16	0.792	0.780	0.745	0.837
13	D-dimer + Mono.CD16	0.811	0.811	0.773	0.845

Model	variables	MEAN	MEDIAN	25%	75%
14	D-dimer + Mono + Mono.CD16	0.824	0.822	0.792	0.864
15	D-dimer + Lactate + Mono.CD16	0.831	0.831	0.779	0.875
16	D-dimer + Lactate + Mono + Mono.CD16	0.839	0.841	0.798	0.880
17	CRP + D-dimer	0.866	0.867	0.856	0.887
18	CRP + Mono	0.872	0.883	0.855	0.894
19	CRP + Lactate	0.872	0.875	0.860	0.890
20	CRP + D-dimer + Lactate	0.874	0.875	0.864	0.890
21	CRP + Lactate + Mono	0.875	0.883	0.860	0.894
22	CRP + D-dimer + Mono	0.883	0.886	0.864	0.902
23	CRP + D-dimer + Lactate + Mono	0.886	0.888	0.867	0.902
24	CRP + Mono.CD16	0.888	0.890	0.867	0.910
25	CRP + Mono + Mono.CD16	0.893	0.890	0.874	0.913
26	CRP + Lactate + Mono.CD16	0.895	0.894	0.871	0.913
27	CRP + D-dimer + Mono.CD16	0.896	0.896	0.871	0.920
28	CRP + Lactate + Mono + Mono.CD16	0.899	0.898	0.883	0.920
29	CRP + D-dimer + Lactate + Mono.CD16	0.900	0.898	0.878	0.924
30	CRP + D-dimer + Mono + Mono.CD16	0.903	0.905	0.886	0.929
31	CRP + D-dimer + Lactate + Mono + Mono.CD16	0.910	0.909	0.890	0.932
32	SeptiScore	0.888	0.888	0.888	0.888
33	SeptiScore + D-dimer	0.893	0.890	0.886	0.898
34	SeptiScore + Lactate	0.895	0.894	0.886	0.902
35	SeptiScore + Mono	0.896	0.898	0.883	0.906
36	SeptiScore + D-dimer + Mono	0.900	0.900	0.890	0.905
37	SeptiScore + CRP	0.901	0.913	0.883	0.917
38	SeptiScore + D-dimer + Lactate	0.903	0.898	0.890	0.909

Model	variables	MEAN	MEDIAN	25%	75%
39	SeptiScore + CRP + Lactate	0.905	0.911	0.886	0.920
40	SeptiScore + Lactate + Mono	0.906	0.909	0.893	0.917
41	SeptiScore + CRP + D-dimer	0.912	0.917	0.902	0.924
42	SeptiScore + D-dimer + Lactate + Mono	0.914	0.909	0.902	0.924
43	SeptiScore + CRP + Mono	0.915	0.913	0.905	0.924
44	SeptiScore + CRP + D-dimer + Lactate	0.915	0.920	0.902	0.925
45	SeptiScore + CRP + Lactate + Mono	0.920	0.924	0.905	0.932
46	SeptiScore + CRP + D-dimer + Mono	0.924	0.922	0.909	0.936
47	SeptiScore + Mono.CD16	0.925	0.920	0.898	0.951
48	SeptiScore + Mono + Mono.CD16	0.929	0.924	0.905	0.952
49	SeptiScore + CRP + D-dimer + Lactate + Mono	0.930	0.932	0.913	0.943
50	SeptiScore + Lactate + Mono.CD16	0.933	0.932	0.902	0.958
51	SeptiScore + CRP + Mono.CD16	0.939	0.939	0.920	0.962
52	SeptiScore + Lactate + Mono + Mono.CD16	0.939	0.943	0.917	0.962
53	SeptiScore + D-dimer + Mono.CD16	0.942	0.943	0.923	0.963
54	SeptiScore + CRP + Lactate + Mono.CD16	0.943	0.943	0.920	0.966
55	SeptiScore + CRP + Mono + Mono.CD16	0.944	0.943	0.924	0.967
56	SeptiScore + D-dimer + Mono + Mono.CD16	0.946	0.947	0.924	0.970
57	SeptiScore + CRP + Lactate + Mono + Mono.CD16	0.948	0.947	0.931	0.970
58	SeptiScore + D-dimer + Lactate + Mono.CD16	0.952	0.955	0.932	0.973
59	SeptiScore + CRP + D-dimer + Mono.CD16	0.952	0.951	0.936	0.973
60	SeptiScore + D-dimer + Lactate + Mono + Mono.CD16	0.956	0.955	0.932	0.974
61	SeptiScore + CRP + D-dimer + Lactate + Mono.CD16	0.956	0.955	0.939	0.973
62	SeptiScore + CRP + D-dimer + Mono + Mono.CD16	0.956	0.955	0.936	0.973
63	SeptiScore + CRP + D-dimer + Lactate + Mono + Mono.CD16	0.961	0.962	0.942	0.978
